# Expression of Heat Shock Protein 70 Gene and Its Correlation with Inflammatory Markers in Essential Hypertension

**DOI:** 10.1371/journal.pone.0151060

**Published:** 2016-03-18

**Authors:** Kamna Srivastava, Rajiv Narang, Jagriti Bhatia, Daman Saluja

**Affiliations:** 1 Dr. B R Ambedkar Center for Biomedical Research, University of Delhi, Delhi-110007, India; 2 Department of Cardiology, All India Institute of Medical Sciences, New Delhi-110029, India; 3 Department of Pharmacology, All India Institute of Medical Sciences, New Delhi- 110029, India; Virginia Commonwealth University, UNITED STATES

## Abstract

**Objectives:**

Hypertension is characterized by systemic high blood pressure and is the most common and important risk factor for the development of cardiovascular diseases. Studies have shown that the circulating levels of certain inflammatory markers such as tumor necrosis factor-alpha (TNF-alpha), interlukin-6 (IL-6), c-reactive protein (CRP), and tumor suppressor protein-53 (p53) are upregulated and are independently associated with essential hypertension. However, mechanism of increase in the levels of HSP70 protein is not clear. No such studies are reported in the blood circulation of patients with essential hypertension. In the present study, we investigated the expression of circulating HSP70 at mRNA and protein levels and its relationship with other inflammatory markers in patients with essential hypertension.

**Materials and Methods:**

We recruited 132 patients with essential hypertension and 132 normal controls from similar socio-economic-geographical background. The expression of HSP70 at mRNA levels was determined by Real Time PCR and at protein levels by indirect Elisa and Western Blot techniques.

**Results:**

We found a significantly higher expression of HSP70 gene expression (approximately 6.45 fold, *P* < 0.0001) in hypertensive patients as compared to healthy controls. A significant difference (*P* < 0.0001) in the protein expression of HSP70 was also observed in plasma of patients as compared to that of controls.

**Conclusion:**

Higher expression of HSP70 is positively correlated with inflammatory markers in patients with essential hypertension and this correlation could play an important role in essential hypertension.

## Introduction

Hypertension is characterized by systemic high blood pressure and is the most common and important risk factor for the development of cardiovascular diseases. In India, the prevalence of adult hypertension has risen dramatically over the past three decades from 5% to 20–40% in urban areas [1]. Epidemiological studies suggest that 20–60% of essential hypertension is inherited and the remaining is acquired or environmental [2].

Essential hypertension acts as a major factor for endothelial dysfunction and vascular damage as it promotes inflammatory activation of endothelial cells in the arterial wall [3]. Recently, it has become evident that the immune system and inflammatory markers such as Interleukin-6, Tumor necrosis factor alpha and C-reactive protein are also essential in the pathogenesis of hypertension. The expression of inflammatory markers was found to be high in tissues such as visceral adipocytes [4], as well as in plasma in hypertensive patients as compared to healthy individuals and are independently associated with essential hypertension [5].

Interleukin-6 (IL-6) is a pro-inflammatory cytokine that is released from numerous cell types, including endothelial cells and vascular smooth muscle cells (VSMCs). IL-6 stimulates the synthesis of C-reactive protein and also promotes VSMC proliferation, a hallmark of hypertension and atherosclerosis [6]. TNF-α is a pro-inflammatory cytokine, stimulates growth of vascular smooth muscle cells (VSMC). Additionally, it increases the expression of adhesion molecules, other cytokines and metalloproteinases. TNF-α antagonist Etanercept reduces the hypertension caused by fructose feeding in rats and prevents the hypertension caused by angiotensin II infusion [7].

CRP is an acute phase reactant produced by hepatocytes whose concentration increases 4 to 6 hours after acute tissue injury or inflammation and declines rapidly with resolution of the injurious process. CRP is considered as an independent predictor of cardiovascular disease and has been associated with increased risk for development of hypertension [8].

Heat shock protein (HSP) act as molecular chaperons playing an important role in normal cell processes. These proteins help in protein folding, assembly, disassembly and translocation of other proteins. These are classified by their molecular weight to which 70 kDa heat shock protein (HSP70) represent a wide family, and are associated with cell protection. Most of our knowledge concerning the regulation and function of HSPs has come from studies of cultured cells [9–11]. Little is known about their expression in vivo, although it is clear that HSPs are induced in rat in response to a variety of stresses [12–14]. Pockley *et al*. have reported the higher circulating levels of HSP70 in patients with established hypertension may have supposed to be future risk of progression of cardiovascular diseases, including atherosclerosis [15].

Genes encoding heat shock proteins are transcriptionally regulated by physiological processes typically associated with cell stress, including the cell cycle, cell proliferation, and differentiation. In human, three genes encoding members of the HSP70 class are mapped within the MHC class III region (6p21.3): HSP70-1 (*HSPA1A*), HSP70-2 (*HSPA1B*) and HSP70-hom (*HSPA1L*). HSP70-1 and HSP70-2 encodes heat-inducible protein HSP70 which differ in their regulatory domains, whereas HSP70-hom encodes a non-heat-inducible form [16]. HSP70 chaperone activity may influence tumorigenesis by regulating the activity of proteins that are involved in cell cycle machinery. HSP70 family members and/or HSP90 transiently associate with key molecules of the cell cycle control systems, including p53 [17–18]. In mammary tumour cell lines, both HSP70 and HSP90 were found to be associated with mutated p53 in the cytoplasm [19]. HSP genes are polymorphic, with some variants responsible for a change in function and susceptibility to stress tolerance [20]. Studies have reported association of HSP70 gene polymorphisms with cardiovascular disease [21–23]. Xu *et al*. have shown that acute hypertension induces a rapid expression of HSP70 mRNA followed by elevated HSP70 proteins in the rat aorta [24]. The HSP70 induction is blocked by prevention of elevation in blood pressure, i.e., administration of the vasodilator agent, sodium nitroprusside. However, it is not known whether HSP70 production is initiated by hemodynamic force per se or by cytokines in vivo. To the best of our knowledge, the expression of circulating HSP70 gene at mRNA and protein levels is not reported so far, in patients with essential hypertension. In the present study, we investigated the expression of circulating HSP70 at mRNA and protein levels in patients with essential hypertension. We have also investigated the correlation of the differential expression of HSP70 with the known inflammatory markers in patients with essential hypertension.

## Materials and Methods

### Study subjects

The study was conducted in 132 unrelated essential hypertensive patients and 132 healthy volunteers as controls in the age range 25–60 years. The sample size was calculated using PS-Power and sample size calculation version 3.0 software, with an α error of 5% and a power of 80% and was found to be adequate in both the study subjects. The study was conducted in accordance with the guidelines of the Helsinki Declaration and written informed consent was taken from each study subjects. Approvals of ethics committee of All India Institute of Medical Sciences, New Delhi (IEC/NP-384/2012) and Dr. B. R. Ambedkar Center for Biomedical Research, University of Delhi (F.50-2/Eth. Com/ACBR/169) were obtained. The study subjects were randomly recruited from the outpatient department (OPD) clinics of hypertension, Department of Cardiology, All India Institute of Medical Sciences, New Delhi, India.

### Inclusion and exclusion criteria of the study subjects

Newly diagnosed hypertensive patients with systolic blood pressure (SBP) more than 140 mmHg and/or diastolic blood pressure (DBP) more than 90 mmHg on two or more consecutive visits were considered as hypertensives. Patients with history of diabetes mellitus, hyperlipidaemia, liver or renal disease, congestive cardiac failure and myocardial infarction were excluded from the study. Patients with pregnancy and lactation and receiving medications for other diseases that could affect blood pressure were also excluded. Blood pressure was measured using Mercury sphygmomanometer and the diagnosis of essential hypertension was based on The Seventh Report of the Joint National Committee on Prevention, Detection, Evaluation, and Treatment of High Blood Pressure (JNC 7) criteria.

The control group was unrelated age and sex matched healthy volunteers. These subjects had no personal or family history of hypertension and other cardiovascular diseases in first-degree relatives and had SBP <130 mmHg and DBP <85 mmHg. Healthy volunteers who visited the outpatient clinics with minor illness without hypertension, diabetes mellitus, hyperlipidaemia and family history of hypertension in previous records were recruited as controls. None of the subjects in the control group was receiving antihypertensive therapy for heart disease or hormone-replacement therapy during the time of the study.

Plasma lipid profile and blood glucose level were measured after overnight fasting in both study groups to rule out diabetes and hyperlipidaemia. All the participants were interviewed using questionnaire with regard to their lifestyle, smoking, alcohol consumption and food intake.

### Sample collection and processing

The peripheral venous blood was drawn between 09:00–11:00 am after an overnight fast, with subjects in seated position and collected in the ethylenediamine tetracetate (EDTA) vial. Five ml of peripheral venous blood was collected from the study subjects, from which one ml of whole blood was used for RNA isolation and the plasma was separated by centrifugation of the remaining volume of blood at 520 x g for 10 min.

### RNA Isolation and Complimentary DNA (cDNA) preparation

Total RNA was isolated from whole blood samples by QIAamp® RNA Blood Mini Kit (QIAGEN®) according to the manufacturer’s protocol. Quantification of RNA was done by Nanodrop (ND-1000). Complimentary DNA (cDNA) was prepared by using First Strand cDNA Synthesis (Fermentas) kit.

### Semi-quantitative PCR

Semi-quantitative PCR for heat shock protein70 (HSP70) gene was performed by using Mastercycler (eppendorf). HSP70 gene expression at mRNA level was quantified by densitometric analysis in terms of integrated densitometry value (IDV) by imageJ software (NIH). The IDV’s for HSP70 gene in study subjects was normalized with 18S rRNA gene expression.

### Real time quantitative PCR

Real time quantitative PCR analysis for HSP70 gene (NM_005527.3) was performed using the following primers: (5’-TTCGTGGCTGGAGGTCAATC-3’, 5’-TAATGATTTGAAGATGAGGGG-3’) and housekeeping gene 18S rRNA (5’-GTGGTGTTGAGGAAAGCAGACA-3’, 5’-TGATCACACGTTCCACCTCATC-3’) in ABI 7300 Real Time PCR System (Applied Biosystem) with SYBR green PCR Core reagents (Eurogentec). All assays were done in triplicate and delta-CT value (the difference between CT values obtained for the gene of interest and normaliser or housekeeping gene), and the difference between the threshold cycle (CT) values obtained for the HSP70 and housekeeping gene 18S rRNA was calculated. The delta-delta-CT equation was used to compare the expression of HSP70 gene between patients and controls in terms of fold difference.

### Isolation of plasma protein

Total protein was extracted from plasma by acetone precipitation method, as described in procedure [25] with slight modifications. Plasma (80 μl) was diluted with 120 μl of 1X Phosphate Buffered Saline (PBS) and mixed with 600 μl of ice cold acetone, kept overnight on 4°C. The sample was centrifuged at 10000 X g, 4°C for 30 min, the supernatant was removed and the pellet was air dried and dissolved in 200 μl of 0.1% SDS. The concentration of total protein was estimated by Bicinchoninic acid (BCA) protein estimation kit (Banglore Genei, India).

### Western blot analysis

Plasma protein (50 μg) was separated on 10% SDS- polyacrylamide gel and transferred to PVDF membrane (MDI, India). The membranes were blocked overnight at 4°C with 3% Bovine Serum Albumin (BSA) and incubated for 3 hrs at room temperature with 1:100 dilution of HSP70 primary antibody (sc-1060, Santa Cruz Biotechnology, USA) and 1:100 dilution of β-actin primary antibody (sc-130656, Santa Cruz Biotechnology, USA) individually, followed by washing (thrice) with Phosphate buffered saline (PBS) containing 0.05% Tween-20. Blots were then incubated with horseradish peroxidase—conjugated secondary antibody of 1:1000 dilution (sc-2357, Santa Cruz Biotechnology, USA) and detected by chemiluminescence detection (WEST-ZOL Plus, Intron Biotechnology, Korea). HSP70 protein expression was quantified by densitometric analysis in terms of integrated densitometric value (IDV).

### Indirect Enzyme-linked immunosorbent assay (iELISA)

The isolated protein was diluted in 0.1M sodium carbonate-bicarbonate (pH 9.4) coating buffer. 100μl of 5 μg/ml protein was coated on flat bottom 96 well microtest plate and incubated for 3 hr at 37°C. After incubation, excess antigen was removed by washing with neutral phosphate buffer saline (PBS). Then wells were blocked over night at 4°C by 3% bovine serum albumin (BSA), incubated for 2 hrs at room temperature with 1:100 dilution of HSP70 primary antibody (sc-1060, Santa Cruz Biotechnology, USA). Any excess antibody was removed by washing with PBS and incubated with horseradish peroxidase—conjugated secondary antibody of 1:1000 dilution (sc-2357, Santa Cruz Biotechnology, USA) for 2 hr. The wells were incubated with 3, 3’, 5, 5’- tetramethyl benzidine (TMB) (eBiosciences lnc. San Dieago, CA, USA) for 30 min at 37°C after washing. The reaction was stopped by 0.2 M sulphuric acid and optical density at 450 nanometre (nm) wavelength was measured by ELISA reader TECAN (Infinite® 200 PRO) and numeric data expressed as relative light unit (RLU), data was expressed as the mean RLU ± SD.

### Enzyme-linked immunosorbent assay (ELISA)

Plasma levels of interlukin-6 (IL-6), tumor necrosis factor-alpha (TNF- alpha), c-reactive protein (CRP), protein-53 (p53) were determined by sandwich-type enzyme-linked immunosorbent assay. Each sample was assayed in triplicate using ELISA Ready-SET-GO e-biosciences, San Diego, USA for Human IL-6 and human TNF alpha, Human CRP kit by Immunology Consultant Laboratory, Portland, USA; Human p53 kit by Boster Biological Technology Abingdon, UK. Optical density at 450 nanometre (nm) wavelength was measured by ELISA reader TECAN (Infinite® 200 PRO) in each assay. The detection limit for Human IL-6, Human TNF alpha, Human CRP and Human p53 was in the range of 02-200pg/mL, 04–500 pg/mL, 1.56–100 ng/mL and 156–10,000 pg/mL respectively.

### Statistical analysis

Statistical analysis was performed using STATA version 12.1 and GraphPad Prism Ver.5 (GraphPad Software Inc., San Diego, CA, USA). The scatter plots were created to show the distribution of individual data in both the study subjects. Differences between the means of the groups were analyzed by chi-square and Student's t-test of significance as appropriate. We have applied multivariate logistic regression analysis regressed for age, sex, systolic and diastolic blood pressure to the data obtained. Correlations between different parameters were determined by Pearson correlation coefficients. P values < 0.05 are considered as significant.

## Results

### Base line characteristics of the study subjects

The hypertensive patients and healthy controls belonged to the same age group, were non-smokers and their lipid profile values such as total cholesterol (TC), triglyceride (TG), high density lipoprotein (HDL) cholesterol and low density lipoprotein (LDL) cholesterol were comparable (Table 1). Systolic blood pressures (SBP) in patients were significantly higher (P = 0.0001) than controls. Similarly, diastolic blood pressures (DBP) in patients were higher (P = 0.0001) than controls.

**Table 1:** Baseline parameters of study subjects.

Parameters	Patients (132) mean (95%CI)^a^	Controls (132) mean (95%CI)^a^	P value*
Age (years)	49.8 ± 12	51.2 ± 6	0.26
Sex (M/F)	80/52	85/47	0.37
Systolic blood pressure	174.69 ± 19.13	116.17 ± 4.25	< 0.0001
Diastolic blood pressure	92.09 ± 12.38	77.25 ± 4.23	< 0.0001
Body mass index(Kg/m2)	25.05 ± 3.54	24.79 ± 2.35	0.80
Heart Rate (Beat/min)	74.2 ± 8.7	71.2 ± 6.2	0.36
Blood Glucose (mg/dl)	91.5 ± 17.6	86.7 ± 15.4	0.87
Blood Urea(mg/dl)	23.8 ± 4.8	21.5 ± 4.2	0.16
Serum Creatinine	1.22 ± 0.3	0.94 ± 0.22	0.22
LDL cholesterol (mg/dl)	86.3 ± 23.4	88.5 ± 29.4	0.27
HDL cholesterol (mg/dl)	45.3 ± 7.2	42.8 ± 7.8	0.13
Total cholesterol (mg/dl)	156.3 ± 41.5	160.3 ± 37.5	0.57
Triglyceride (mg/dl)	150.0 ± 44	144.0 ± 35	0.36

Data are means ± SD, Means values of the different parameters in the study subjects were analyzed by chi-square and Student's *t*-test of significance as appropriate

^a^95% confidence interval, LDL High-density lipoprotein, LDL Low-density lipoprotein

*Patients vs Controls. P < 0.05 is considered significant.

### Heat shock protein 70 (HSP70) gene expression

**Fig 1:**
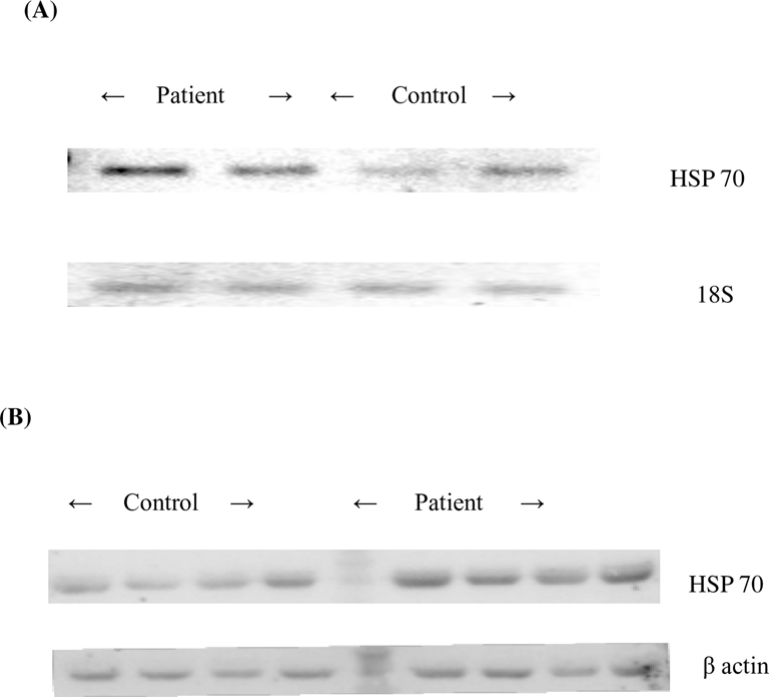
Semi-quantitative gel picture and Western blot analysis of Heat shock protein70. (A) represents the semi-quantitative analysis of heat shock protein70 (HSP70) gene and 18S rRNA (Housekeeping gene) in controls and patients respectively.(B) represents the western blots of heat shock protein70 (HSP70) and β-actin protein in controls and patients respectively.

**Fig 2:**
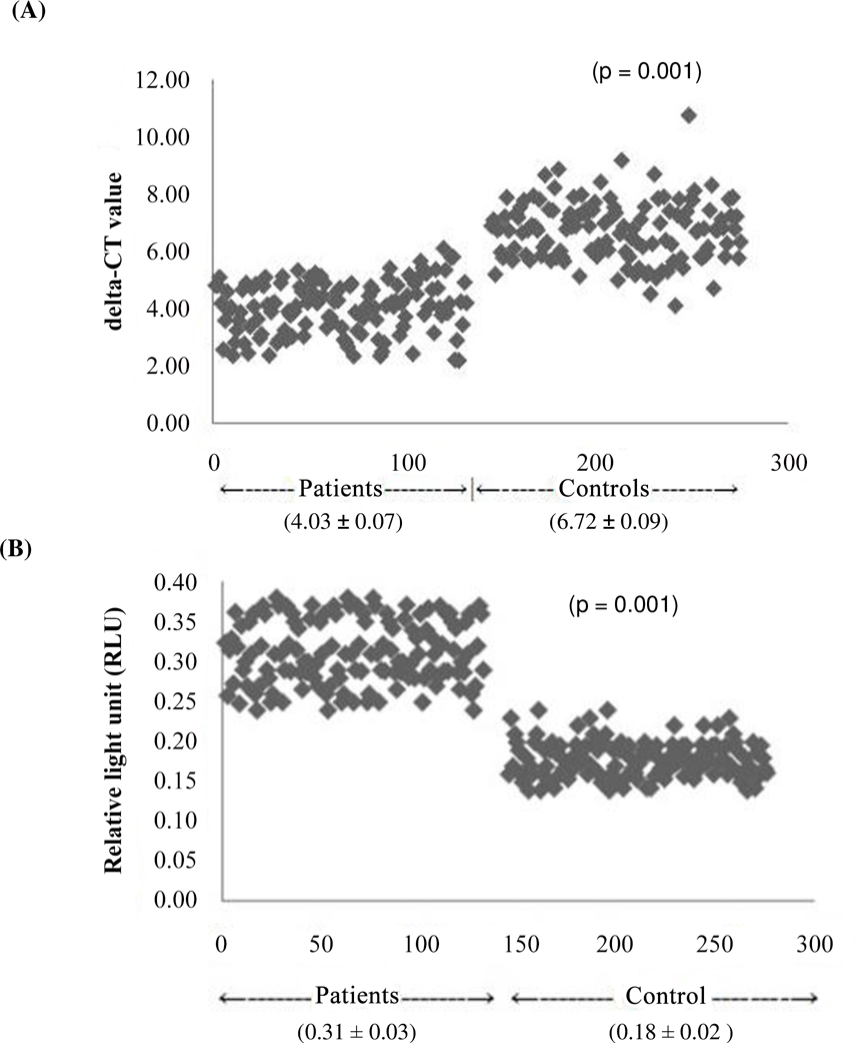
qRT-PCR analysis of Heat shock protein70 gene expressions at mRNA level and indirect ELISA analysis at protein level. (A) represents the scattered plot of levels of HSP70 gene expression at mRNA level in the individual study subjects by qRT-PCR. The relative HSP70 gene expression in patients and controls is expressed in delta-CT values. Results were expressed as densitometric ratio (HSP70 to 18S rRNA gene) in patients and controls group ± SD. P < 0.001. Higher delta-CT value deflects the lower gene expression. (B) represents the scattered plot of individual data of HSP70 expression at protein level by indirect ELISA technique in the study subjects. P < 0.0001.

**Fig 3:**
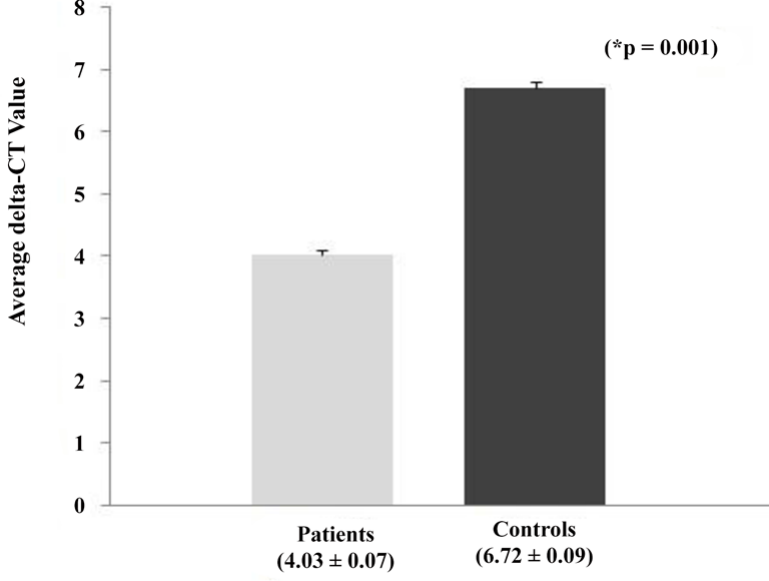
Heat shock protein70 mRNA expression in study subjects. Fig 3 represents the expression of HSP 70 at mRNA levels in study subjects. Results were expressed as average delta-CT value in patients and controls group ± SD. P < 0.0001 is considered to be significant.

The expression of HSP70 at mRNA levels in 132 patients and 132 controls are carried out by RT-PCR. Semi-quantitative analysis of HSP70 gene expression was determined in 26 patients and 26 controls by calculating the integrated densitometric value (IDV) through densitometry. The HSP70 gene expression in terms of average IDV normalized to 18S rRNA gene expression in patients and controls were 0.92 ± 0.12 and 0.73 ± 0.12 respectively (Fig 1A). To get a better insight, real time qPCR was carried out for all the subject samples. Quantitative analysis of HSP70 gene expression was calculated by delta-delta-CT method. Fig 2A represents the individual delta-CT value of study subjects. The average delta-CT value for patients and controls were found to be 4.03 ± 0.07 and 6.72 ± 0.09, respectively, and the difference in the delta-CT values were found to be statistically significant (P < 0.001) **(Fig 3).** The relative expression of mRNA for HSP70 gene to 18S rRNA in patients with essential hypertension was increased by approximately 6.45 fold as compared to control. The higher delta-CT value represents lower expression of gene at mRNA level and vice-versa.

### Heat shock protein 70 (HSP70) expression by Western blot

Fig 1B shows a representative western blot of plasma HSP70 and β-actin protein of 132 patients with essential hypertension and 132 controls. A significant difference for the HSP70 plasma protein expression level was observed in patients (1.43 ± 0.02) and control groups (1.11 ± 0.01) (P < 0.0001). The HSP70 protein level was found to be 1.3 times higher in patients as compared to that of controls.

### Heat shock protein 70 (HSP70) expression by Indirect ELISA

HSP70 expression at protein level by indirect ELISA method in 132 patients with essential hypertension (0.31 ± 0.03 RLU) and 132 healthy control groups (0.18 ± 0.02 RLU) (P < 0.0001) and the protein level was found to be 1.7 times higher in patients as compared to that of controls (Fig 2B).

### Inflammatory markers in study subjects

**Fig 4:**
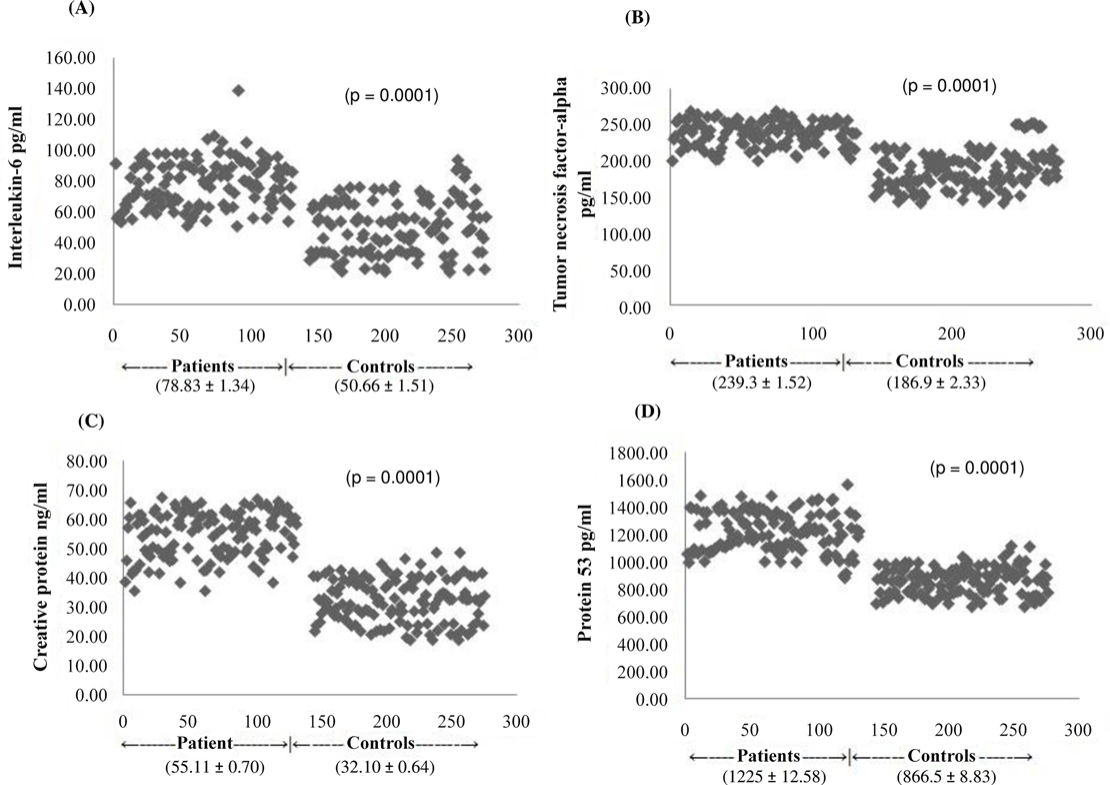
Plasma levels of inflammatory markers. (A) represents the scattered plot of levels of interleukin-6 in the individual study subjects. (B) represents the scattered plot of individual data of tumor necrosis factor-alpha in the study subjects. (C) represents the scattered plot of individual data of C-reactive protein in the study subjects. (D) represents the scattered plot of individual data of p53 in the study subjects.

Table 2 represents the plasma levels of inflammatory markers in the study subjects. Mean values of concentration of inflammatory markers such as IL-6, TNF-alpha, CRP and p53 levels were significantly higher in patients as compared to controls group (Fig 4).

**Table 2:** Plasma levels of inflammatory markers.

Groups	Mean values of concentration of inflammatory markers, pg/ml			
	IL-6	TNF-alpha	CRP	p53
Patient (n = 132)	78.83 ± 1.34	239.3 ± 1.52	#55.11 ± 0.70	1225 ± 12.58
Control (n = 132)	50.66 ± 1.51	186.9 ± 2.33	#32.10 ± 0.64	866.5 ± 8.83
P-values	0.0001	0.0001	0.0001	0.0001

Data are means ± SD

^#^ concentration in ng/ml, Mean value of the different

Parameters in the patients and controls were analyzed by Student's *t*-test of significance

P < 0.05 is considered significant.

### Correlations between study parameters

Table 3 represents the correlation study between different parameters in the study subjects by Pearson correlation coefficients. There were significant positive correlations between the circulating HSP70 gene expression (delta-CT) values and different circulating inflammatory markers such as IL-6, TNF-alpha, CRP and p53 levels in patients with essential hypertension. In the same group positive correlations were observed among circulating inflammatory markers. However, no such correlations were observed in control group for different study parameters except with TNF-alpha and p53. We investigated whether inflammatory markers (IL-6, TNF-alpha, CRP, and p53) of essential hypertension are related to circulating HSP70 gene expression by ELISA as described under methods. The correlation of expression of HSP70 protein with other markers was calculated by Pearson correlation coefficients. There were significant negative correlations between the delta-CT values and different circulating inflammatory markers such as IL-6, TNF-alpha, CRP and p53 levels in patients with essential hypertension. In the same group positive correlations were observed among circulating inflammatory markers. However, no such correlations were observed in control group for different study parameters except with TNF-alpha and p53 (r = 0.18, P = 0.04). The expression of HSP 70 at protein levels in terms of IDV was positively correlated with the different inflammatory markers (IL-6, TNF-alpha, CRP, and p53) in patients group. There was no significant difference observed for the correlation between different inflammatory markers and IDV for controls group. We observed a statistically significant negative correlation between delta-CT value and IDV for the patients group. However, no significant difference was observed for the correlation between delta-CT value and IDV in controls group (Table 3).

**Table 3:** Correlation study between different parameters in the study subjects.

Parameters	Correlation coefficient			
	Patients		Controls	
	r	*P value*	r	*P value*
IL-6 and delta-CT	-0.5	0.003	0.03	0.8
TNF-alpha and delta-CT	-0.59	0.0001	0.13	0.15
CRP and delta-CT	-0.67	0.0001	0.05	0.5
p53 and delta-CT	-0.65	0.0007	0.16	0.06
IL-6 and TNF-alpha	0.48	0.0001	-0.01	0.9
IL-6 and CRP	0.56	0.0001	0.07	0.4
IL-6 and p53	0.54	0.001	0.05	0.5
TNF-alpha and CRP	0.65	0.0001	-0.01	0.9
TNF-alpha and p53	0.66	0.0001	0.18	0.04
CRP and p53	0.72	0.001	0.1	0.23
IL-6 and IDV	0.55	0.001	0.18	0.72
TNF-alpha and IDV	0.45	0.0001	0.15	0.17
CRP and IDV	0.64	0.0001	0.08	0.32
p53 and IDV	0.75	0.0001	0.06	0.28
delta-CT and IDV	-0.52	0.0001	-0.23	0.13

CRP C-reactive protein, IL Interleukin, TNF tumor necrosis factor, p53 Tumor suppressor protein-53, CT Threshold cycle, r = Pearson correlation, IDV integrated densitometry value; P < 0.05 is considered significant.

### Multivariate logistic regression analysis

We have applied multivariate logistic regression analysis regressed for age, sex, systolic and diastolic blood pressure to our data obtained. All the parameters, such as HSP70 gene and protein expression, IL-6, TNF-alpha, CRP and p53 were highly correlated so omitted in the multivariate analysis and exist as independent risk factor in patients with essential hypertension (Table 4).

**Table 4:** Multivariate logistic regression analysis of different parameters in the study subjects.

Parameters	Group	Odds Ratio	[95% Confidence Interval Lower Higher		P- value
**IL6**	Patient/Control	11.72532	6.034892	22.78138	0.000
**TNF-alpha**	Patient/Control	42.04612	19.25059	91.83491	0.000
**CRP**	Patient/Control	146.9205	55.97541	385.6272	0.000
**p53**	Patient/Control	117.1727	34.15457	401.9798	0.000
**RT-PCR**	Patient/Control	178.4622	61.69645	516.2168	0.000

CRP C-reactive protein, IL Interleukin, TNF tumor necrosis factor

p53 Tumor suppressor protein-53, CT Threshold cycle

Regressed for age, sex, systolic blood pressure and diastolic blood pressure

*P* < 0.05 is considered significant

## Discussion

Several epidemiological and clinical studies demonstrated a relationship between Heat Shock Protein 70 and hypertension [15, 22, 26]. Essential hypertension is a common health disorder with uncertain etiology and unclear pathophysiology. There are reports suggesting the association of HSP70 gene with cardiovascular diseases including essential hypertension [21–23]. In the present study, we provide the first evidence that essential hypertension induces circulating expression of HSP70 at mRNA and protein levels and its interrelationship with plasma inflammatory markers.

The involvement of pro-inflammatory processes in cardiovascular disease such as hypertension is well accepted. The evaluation of inflammatory markers and cytokines are better approaches to understand the pathogenesis of cardiovascular diseases, which is helpful to reduce the emerging risk of essential hypertension [27]. Some cross-sectional studies showed that plasma levels of inflammatory markers, such as C- reactive protein (CRP) and cytokines [TNF-alpha (tumor necrosis factor-alpha) and IL-6 (interleukin-6)] as well as adhesion molecules are increased in patients with essential hypertension as compared to healthy individuals supporting the role of inflammation in the pathogenesis of hypertension [28–31]. Asea *et al*. reported the dual role of HSP70, both as a chaperone in extracellular and as cytokine in intracellular milieu [32]. In contrast, an in-vitro study suggests that fragments from HSP70 and HSP60 are immunogenic and can stimulate macrophages to release pro-inflammatory cytokines such as TNF alpha [33]. The variation in the levels of inflammatory markers was correlated with change in blood pressure levels, considered as a key factor for their association with hypertension and target organ damage [34]. Our study also suggests an increase in the levels of circulating cytokines such as IL-6, TNF-alpha and CRP in patients and is in agreement with the previous studies [28–31]. Krause and co-workers (2015) suggested that there is a divergence between the concentration of extracellular HSP70 and its intracellular concentration in disease conditions such as inflammation Driven Type 2 Diabetes [35]. They further suggested that the ratio of extracellular HSP70 to intracellular HSP70 represent a better marker for inflammation related diseases such as cardiovascular disorders. However, detailed mechanism and role of HSP70 in essential hypertension needs further investigation.

Elevated CRP level may lead to total stroke and hypertension [36]. Also increased CRP levels are one of the major causes of pre-hypertension, which may be considered as short-term risk for ischemic stroke [37]. Bomfin *et al*. demonstrated that the CRP is able to stimulate IL-6 and TNF-alpha production in vascular smooth muscle cells via NFκB and MyD88- independent TLR4 signaling pathway [38]. Higher levels of CRP may increase blood pressure by reducing nitric oxide production in endothelial cells, resulting in vasoconstriction and increased production of endothelin-1 which are the markers for endothelial dysfunction [39]. CRP effectively predicts future hypertension [40–41], suggesting that inflammation may precede and not just follow blood pressure elevation. Our study affirms the association of C-reactive protein in essential hypertension as is evident from the higher levels of CRP in patients as compared to that of controls. Abramson *et al*. (2006) also showed positive correlation of the inflammatory markers, such as CRP and TNF-α with rise in blood pressure [42]. In our study, a positive correlation was observed between CRP and TNF-alpha. Taken together, it suggests that CRP and TNF-α may be one of the factors that predisposes individuals to hypertension.

Although, role of IL-6, TNF-alpha, CRP are much debated, circulating levels of Tumor suppressor protein, p53 is less investigated in patients with essential hypertension. We have found significant increase in the plasma levels of p53 in patients with essential hypertension as compared to controls. Whether the increase in plasma p53 was the cause or the effect of inflammation is not clear at present. A highly significant positive correlation of plasma levels of p53 with IL-6, TNF-alpha and CRP in patients was observed in our study. However, this correlation was not observed in control group except between TNF—alpha and p53 levels.

The endothelium is the organ targeted by metabolic risk factors for CVD and may be the origin of vascular reaction [43]. Stefandi *et al*. [27] reported that inflammatory process and endothelial dysfunction play a fundamental role in the pathogenesis and progression of hypertension. A previous study by Srivastava *et al*. [44] validated a correlation of plasma markers of endothelial dysfunction and further suggested that higher levels of sVCAM-1 and E- selectin and lower levels of Nitric oxide are associated with essential hypertension. Miller *et al*. [45] reported that plasma level of sE-selectin was significantly associated with blood pressure in Women. Elevated blood pressure is a mechanical stress to the endothelium inducing enhanced expression of Heat Shock Proteins on the endothelial cell surface [46]. Although heat shock (stress) proteins are typically regarded as being exclusively intracellular molecules, it is now apparent that they can be released from a variety of viable (non-necrotic) mammalian cells, including endothelial cells [47–49]. HSP60 and HSP70 are present in the sera of clinically normal individuals [11, 26]. Inherent anti-inflammatory properties of HSP70 and its effects on biology and functional status of endothelial cells have been suggested [50]. Animal models have revealed a relationship between elevated blood pressure and increased expression of HSP. Xu *et al*. reported that HSP70 induction occurs as a physiological response to acute hypertension and suggested the possibility that HSP70 plays a role in protecting the vasculature from damage during hemodynamic stress [24]. However, an in-vivo study by Sadoshima *et al*. [51] showed lack of stretch induced expression of HSP70 gene in culture cardiac myocytes suggesting that other factors or subsequent events may be needed for HSP70 induction in the process of cell stretching. Majority of these studies on altered HSP70 expression are pertaining to animal and cell culture studies. We in this study, observed a significant increase in the expression of HSP 70 at mRNA and protein levels in patients with essential hypertension.

We found approximately 6 fold up-regulation of HSP70 gene expression in patients with essential hypertension in comparison to normal controls. Similarly, we also observed a significant increase (1.3 fold) in the expression of HSP70 gene at protein level in patients with essential hypertension as compared to controls which are well supported by the studies indicating the role of HSP70 in the regulation of blood pressure in animal and cell culture studies [24, 51].

The present study has certain limitations. Several important biomarkers of endothelial dysfunction such as E-selectin, sVCAM-1 and nitric oxide were not investigated in plasma samples of patients and controls. However, in a previous study, we reported higher levels of plasma E-selectin, sVCAM-1 and lower levels of nitric oxide in patients with essential hypertension as compared to normal controls [44].

## Conclusion

We, for the first time report higher expression of HSP70 gene is correlated with circulating levels of inflammatory markers in patients with essential hypertension. It appears that there is a cross talk between the HSP70 and proinflammatory cytokines. In our study, HSP70 gene and protein expression and inflammatory markers such as IL-6, TNF-alpha, CRP and p53, emerged as independent risk factors. However, studies are required to determine whether circulating HSP70 gene plays a causative role in the pathogenesis of essential hypertension or it is one of the consequences of the disease. Further follow up studies are also needed to explore whether up-regulated HSP70 gene expression is an independent risk factor and can it serve as prognostic marker for essential hypertension. We envisage that studies in this direction may lead to better insight into the role of higher expression of HSP70 in essential hypertension.
